# The First Dorsal Metatarsal Artery Perforator Flap: A Description and Anatomical Study

**DOI:** 10.3390/jcm14124136

**Published:** 2025-06-11

**Authors:** Mathilde Saboye, Alexis Majchrzak, Grégoire d’Andréa, Nicolas Bronsard, Olivier Camuzard, Elise Lupon

**Affiliations:** 1 Institut Universitaire Locomoteur et du Sport, Pasteur 2 Hospital, University Côte d’Azur, 06100 Nice, France; mathilde.saboye@umedicina.cat (M.S.); alexis.majchrzak@etu.unice.fr (A.M.); bronsard.n@chu-nice.fr (N.B.); camuzard.o@chu-nice.fr (O.C.); 2 Facultat de Medicina UVic-UCC, University of Vic-Central University of Catalonia, 08500 Barcelona, Spain; 3 Ear, Nose, and Throat Department, University Hospital Center of Nice, 06003 Nice, France; dandrea.g@chu-nice.fr; 4 UR2CA—Unité de Recherche Clinique Côte d’Azur, University Côte d’Azur, 06100 Nice, France; 5 Laboratory of Molecular PhysioMedicine (LP2M), UMR 7370, CNRS, University Côte d’Azur, 06100 Nice, France

**Keywords:** first dorsal metatarsal artery perforator flap, perforator flap, greater toe coverage, acral melanoma reconstruction, anatomical study, hallux soft-tissue reconstruction

## Abstract

**Background/Objectives:** Soft-tissue defects surrounding the big toe can be a challenging problem for reconstructive surgeons. The first dorsal metatarsal artery (FDMtA) arises from the dorsalis pedis artery, which itself arises in front of the ankle joint from the anterior tibial artery. This study aimed to characterize the FDMtA cutaneous perforators (FDMtAPs) and evaluate the potential of a local pedicled perforator flap to cover a hallux soft-tissue defect. **Methods:** Nine feet from fresh cadavers were dissected to describe the FDMtAP anatomy. For each artery, we recorded the origin’s position from the FDMtA, the proximal and distal diameters, and the artery’s course, length, number, and type. We described the FDMtA perforator flap harvest and its application through a clinical case. **Results:** A mean of 3.67 ± 1.23 FDMtAPs were found from the nine dissected feet, with at least 2 perforators per foot. Around 88% were located between 0 and 4 cm along the axis at their origin from the FDMtA, with an area of around 8 cm^2^ and a mean of 2.35 ± 0.36 cm long. The proximal diameter had a mean of 0.178 ± 0.037 mm and 0.110 ± 0.008 mm at the distal diameter. A pedicled flap was readily feasible for all dissections. The case described had satisfactory healing, correct functional, and aesthetic recovery at two months. **Conclusions:** The first dorsal metatarsal artery perforator flap seems to be a reliable and valuable solution for the hallux soft-tissue reconstruction, notably after the excision of acral melanoma.

## 1. Introduction

Soft-tissue defects surrounding the big toe can be a challenging problem for reconstructive surgeons [1]. Alongside traumatic etiologies, oncological causes are frequent sources of greater toe soft-tissue defects. Acral lentiginous melanoma of the hallux remains challenging to identify due to its sometimes hidden location on the foot [2]. Besides the fact that it is the least common subtype, comprising 2 to 3% of all melanomas, it is also the most common diagnosis on the lower limb. Frequently diagnosed at a later stage, surgery becomes more mutilating, leading to a significant degree of morbidity and mortality [3]. Current management requires a large excision of the adjacent tissue of the initial lesion, notably the nail matrix. It is, therefore, essential to identify the best means of repairing this anatomical zone to enable functional and aesthetic recovery of the big toe and improve patients’ quality of life.

Indeed, although the existing literature and anatomical studies on dorsal foot vascularization [4,5,6] provide valuable guidance for surgical planning, they remain limited when it comes to the detailed study of foot perforators. The dorsal foot vascularization is provided by the dorsal pedis artery (DPA), a continuation of the anterior tibial artery (ATA), emerging after crossing the inferior extensor retinaculum. It runs between the tendon of the extensor hallucis and the first tendon of the extensor digitorum longus. The DPA will end in the first intermetatarsal space into two arteries: the deep plantar artery and the first dorsal metatarsal artery (FDMtA), the only metatarsal artery coming from the DPA.

The FDMtA will course in the first interosseous space, presenting an anastomosis with the plantar circulation by the distal artery perforator. Then, it will end its course into two divisions: the hallux’s lateral dorsal digital artery and the second toe’s medial dorsal artery, while having an anastomotic connection with the plantar arterial network. This current knowledge of dorsal foot vascularization has led to innovations over the last ten years in developing new techniques for repairing defects in the dorsum of the foot [7,8,9,10,11]. However, if local/regional flaps could allow a correct reconstruction of the greater toe soft-tissue defect, it often includes extended donor site morbidity [12,13], risk of complications [8,10], and limited reach [9,10].

The Tokyo Consensus proposed a definition and a classification of the propeller flap in 2009. It was defined as an “island flap that reaches the recipient site through an axial rotation” [14]. They first described three types: the subcutaneous pedicled propeller flap, the perforator pedicled propeller flap, and the supercharged propeller flap. Afterward, the axial pedicled propeller flaps category was added to the consensus in 2017. The muscle propeller flap and the chimeric propeller flap were finally added in 2020 [15]. The flap described in this study belongs to the category of pedicled propeller flaps and is referred to as the first dorsal metatarsal artery perforator flap (FDMtAP flap).

The FDMtAP flap remains poorly described in the literature. The limited anatomical characterization of the cutaneous perforators arising from the first dorsal metatarsal artery (FDMtA) [16,17,18,19] hinders the optimal use of this propeller flap in clinical practice.

Therefore, this study aims to assess the value of using a dorsal pedicled intermetatarsal flap based on the cutaneous perforator’s arteries from the FDMtA. We performed an anatomical study on seven cadaveric specimens. We reported the characteristics of the cutaneous dorsal distribution of the foot supply by the first dorsal metatarsal artery perforators (FDMtAPs). We finally provide an application through a clinical case.

## 2. Materials and Methods

### 2.1. FDMtA Perforator Mapping

The anatomical study consisted of the dissection of nine feet from fresh cadavers. The Anatomy Laboratory of the Faculty of Medicine of Nice, France, generously provided the specimens and material used for the study. The French National Ethics Committee approved this study, and it was conducted following the Helsinki Declaration.

The nine feet were dissected under a microscope (×7, Zeiss OPMI Pentero, Carl Zeiss Meditec AG, Jena, Germany) to study the cutaneous perforating arteries of the first dorsal metatarsal artery of the foot (FDMtAP). To begin with, approximately 48 to 72 h before dissection, the pedal arteries were dissected in their sub-retinacular portion (inferior retinaculum of the extensors of the foot), leaving the territory of the FDMtA intact. Depending on the subject, they were then injected with 4 cm^3^ of red latex to stain the FDMtA and its perforators. The cadavers were then stored supine at 4 °C until dissection.

**Figure 1:**
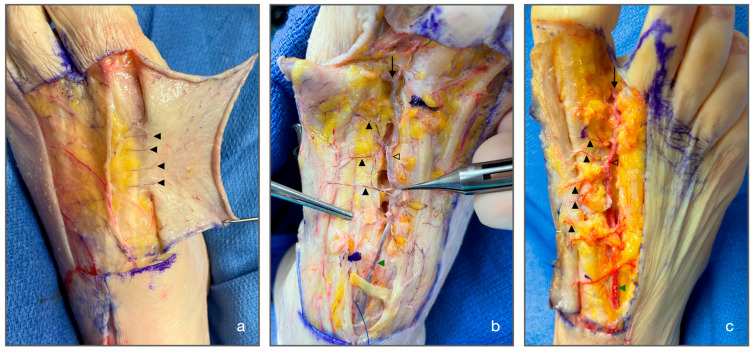
Pictures of three cadaveric dissections allowing the correct visualization of the FDMtA and cutaneous FDMtAPs. (**a**) Subcutaneous plan: We can identify four superficial FDMtAP (▲) ending in the skin (**b**) Adipose plan: The DPA (▲) and the FDMtA (△) have been dissected. Black triangles indicate the beginning of three FDMtAPs. The black arrow shows the distal division into two dorsal digital arteries. (**c**) Adipose plan: The same structures and divisions are recognizable as in image (**b**), but with five FDMtAPs (▲). ▲ = cutaneous FDMtAP; △ = FDMtA; ▲ = DPA; ↓ = dorsal digital artery. FDMtA: first dorsal metatarsal artery; FDMtAPs: first dorsal metatarsal artery perforators; DPA: dorsal pedis artery.

The dissection of the FDMtA began with a scalpel incision using an n°11 cold blade aimed at reclining the cutaneous plane independently of the subcutaneous cellular plane so as not to damage the cutaneous perforators of the FDMtA. The artery was dissected from its origin at the dorsal pedis artery (DPA) to its termination behind the interdigital commissure via the dorsal digital arteries of the first and second toes and a perforating branch with the corresponding plantar pedicle interosseous artery. It was then possible to dissect the cutaneous perforating arteries along the path of the FDMtA to the skin (Figure 1).

We counted and mapped their distribution. Using a caliper (vernier caliper, Fowler High Precision, Newton, MA, USA), measurements of the caliber of the FDMtA and its cutaneous perforators were taken (in millimeters). In this way, we obtained each of their proximal and distal diameters and an average. We also measured the length (in centimeters) of the FDMtA and its perforators using a graduated ruler and their position relative to the axis of the foot’s first metatarsal (from the head to the base of the metatarsal).

### 2.2. Clinical Application

We presented a clinical case to cover a skin defect in a patient with acral melanoma.

**Figure 2:**
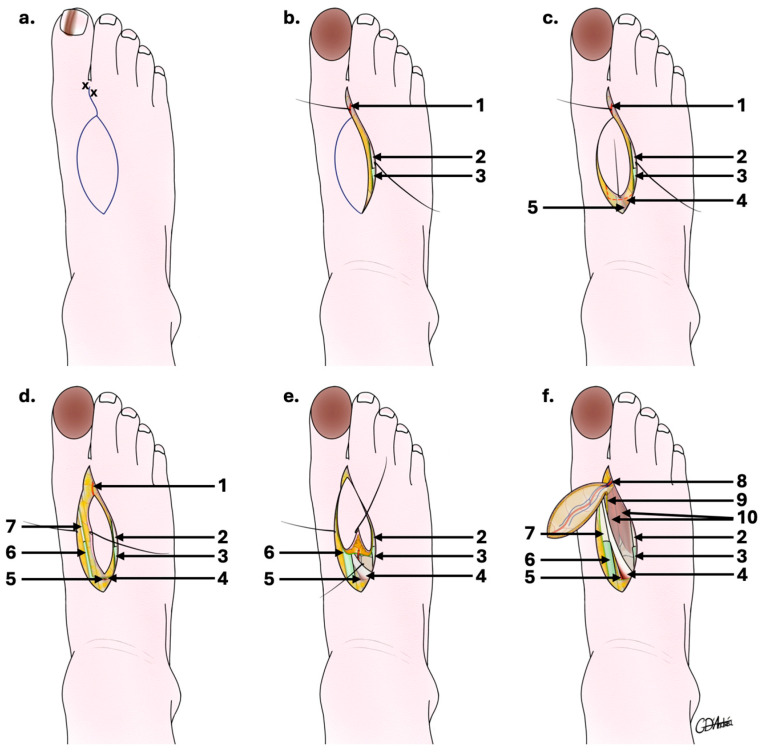
Schematic outline of the first dorsal metatarsal artery perforator flap surgical steps. (**a**) Drawing of the flap after Doppler marking; (**b**) fibular flap edge first incision enabling FDMtA to be located distally; (**c**) continued incision of proximal flap edges enabling FDMtA to be located proximally; (**d**) tibial flap edge incision follows the same depth but includes the peritendon of the extensor hallucis longus; (**e**) ligation of the FDMtA proximally and retrograde dissection; (**f**) harvested FdMtA flap with distal FDMtA dissection allowing a 180° flap rotation. 1: distal part of the first dorsal metatarsal artery; 2: tendon of the extensor muscle of the second toe; 3: synovial sheath of the extensor muscle of the second toe; 4: short extensor hallux muscle; 5: proximal part of the first dorsal metatarsal artery; 6: synovial sheath of the long extensor muscle of the hallux; 7: tendon of the long extensor muscle of the hallux; 8: distal part of the first dorsal metatarsal artery in the flap; 9: medial branch of the deep fibular nerve; 10: first dorsal interosseous muscle of the foot.

The surgical procedure is schematically illustrated in Figure 2.

At the pre-surgical evaluation, the FDMtA and its principal perforators were located using a sound Doppler. An elliptical flap drawing on the foot’s dorsal side was made according to the dimensions expected to cover the exact area that would be excised (Figure 2a).

The procedure was performed under general anesthesia for a duration of approximately 90 min, including 30–45 min for flap removal. First, the cancerous nail matrix of the hallux was removed with skin margins extending from 6 to 10 mm, with bone recut at the dorsal surface of the distal phalanx (P2). A first incision was made at the level of the first interdigital space on the dorsal face of the foot, itself delimited medially by the tendon of the long extensor of the hallux and laterally by the extensor of the second toe (Figure 2b).

An incision was made on the fibular side of the flap, enabling the FDMtA to be located proximally (Figure 2c). The incision depth included part of the peritendon of the extensor digitorum of the second toe. A second tibial incision of the flap follows the same depth but includes the peritendon of the extensor hallucis longus (Figure 2d).

When well individualized, the FDMtA was proximally clipped, and a retrograde dissection of the FDMtA down to the peritendon region was performed, respecting the medial branch of the deep fibular nerve (Figure 2e). This allows localization of the emergence of the most distal cutaneous perforating artery and sectioning of the FDMtA perforators proximal to it. The flap was reclined from proximal to distal until the last distal cutaneous perforator (Figure 2f) and its anastomoses opposite the neck of the first metatarsal were visible. The island flap was accompanied by its vascular pedicle, formed by the last cutaneous perforating artery, lifted, rotated to 180°, and advanced to cover the defect from the hallux to P2. A graft was not indicated to be performed to close the donor site, as the surface area is relatively small. We therefore recommend using an artificial dermis (in this case, Integra^TM^, a two-layer synthetic skin and acellular dermal substitute) or only suturing with 5.0 monofilament. The artificial dermis was placed over the tissue defect of the donor site and sutured, encouraging the cell regeneration process and providing protection against infection.

The patient was followed up on days D1, D14, and D30 and at 16 months.

## 3. Results

### 3.1. Anatomical Description

**Figure 3:**
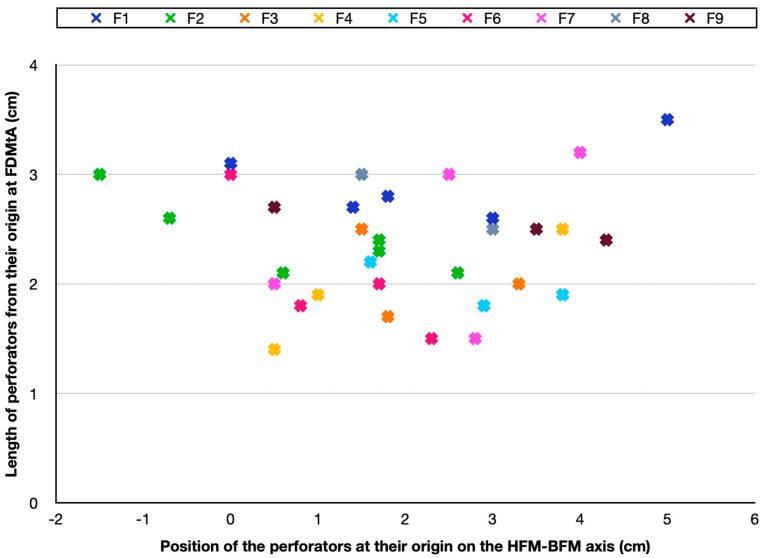
Cartography of skin arteries perforators position of the nine feet studied in the HFM-BFM axis. A cross of a different color represents each foot. Every cross indicates the position of the FDMtAP in the HFM-BFM axis of each foot. It also shows the length (in centimeters) from their origin in the FDMtA. FDMtA: first dorsal metatarsal artery; FDMtAPs: first dorsal metatarsal artery perforators. HFM: head of the first metatarsal; BFM: base of the first metatarsal; F1 = foot n°1; F2 = foot n°2.

The measurement of skin perforators enabled us to produce a cartography of the nine feet studied, exposed in Figure 3.

To highlight their distribution, they were described along an axis corresponding to the distance between the head of the first metatarsal and the base of the first metatarsal (HFM-BFM axis). Around 88% (29/33 perforators) of cutaneous perforators were found between 0 and 4 cm along the axis at their origin from the FDMtA. They were located over an area of around 8 cm^2^ (2 × 4) along the axis. It totaled 33 perforators on the nine feet.

In Table 1, we grouped the results of the FDMtAP analysis. On the nine feet studied, we found a minimum of two cutaneous perforators and a maximum of six for one of them (Table 1: foot n°2). The average was 3.67 perforators per foot (SD ± 1.23). The mean length was about 2.35 cm (SD ± 0.36) from their origin at the FDMtA. The proximal diameter had a mean of 0.178 mm (SD ± 0.037), and the distal diameter had a mean of 0.110 mm (SD ± 0.008).

### 3.2. Clinical Application

**Figure 4:**
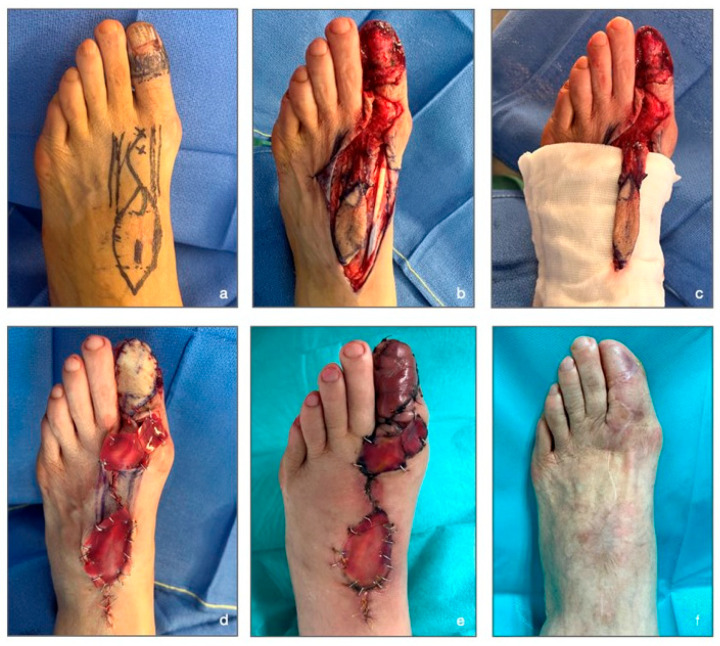
Illustrative case report. (**a**) Pre-surgery: drawing of the flap on the donor site and marking of the FDMtA perforator artery localization with crosses. (**b**) Intraoperative stage: flap dissection and ungual apparatus removal. (**c**) Intraoperative stage: flap and pedicle isolation. (**d**) Immediate post-surgery stage: application of a dermal matrix on the donor site and the flap is sutured. (**e**) 13 days post-surgery: the flap started to heal, peripherical edema visible. (**f**) 16 months post-surgery: final result and the complete healing process.

Based on this study, we describe an original case from our department in which we utilized the FDMtA perforator flap to achieve greater toe coverage (Figure 4).

A 74-year-old female presented acro-lentiginous melanoma of the greater toe on the left foot. The anatomopathological assessment of the pre-operative biopsy revealed the presence of melanocytic proliferation, with Breslow indices of less than 1 mm and affectation of the subepidermal matrix. The IHC PRAME (immunohistochemistry preferentially expressed antigen in melanoma) also demonstrated the existence of over 75% melanocytic components in the biopsy specimen. The pTNM stage study (anatomopathological examination) showed the patient was at stage Tis (tumor in situ). In Figure 4a (pre-surgery), we can observe the pre-surgical drawing of the flap and the hallux margin removal. Figure 4b,c (intraoperative stages) reveal the extended nail resection, including the sub-ungual matrix. The flap harvest with his pedicle, constituted by the FDMtA, is shown in Figure 4c.

The donor site was covered with an Integra™, and the FDMtA flap was laid to recover the soft-tissue defect after the melanoma excision on the hallux. The immediate post-surgery results are shown in Figure 4d. The patient was discharged from the hospital on day one. Greasy dressings were applied every two days by a nurse at home. On post-operative day 14, the patient was pain-free, and sutures were removed. We constated the flap viability despite the flap’s reddish appearance and the dermal matrix adhesion. The patient reported minimal peripheral edema in the toes and foot. After 1 month, the patient was completely healed.

The last clinical follow-up, at 16 months, showed no complications (Figure 4f). The first toe presented a normal range of motion at clinical examination. The nail apparatus has been entirely covered with the flap. Every space filled in by the dermal matrix had healed without a skin graft, and the patient expressed satisfaction regarding the foot’s aesthetic aspect.

## 4. Discussion

The essential role of the foot in enabling walking and good balance makes it vital to respect the functional aspect of reconstructive surgery. It should be remembered that until a few years ago, digital amputation was the procedure of choice for patients with subungual melanoma of the big toe to ensure sufficient excision margins [20]. The consequence was a reduction in function that affected patients’ daily activities. The aesthetic aspect was, therefore, less satisfactory. It has been shown that the survival rate of these patients does not depend on the grade of excision or amputation but on the stage at which the melanoma is diagnosed [20].

The surgeon’s challenge is obtaining complete carcinologic excision margins with a correct functional and aesthetic post-operative outcome. Based on the Breslow index, the recommended excision depth for acro-lentiginous melanoma is suggested by the American Joint Committee on Cancer (AJCC) [21]. At stage Tis (in situ lesion), the clinical margin should be from 0.5 to 1 cm. For the other stages, a surgical margin of 1 cm for T1 (Breslow < 1.0 mm), 1 to 2 cm for T2 (Breslow from 1.1 to 2.0 mm), and more than 2 cm margin for T3 and T4 (Breslow > 2.1 mm) is recommended [21]. However, if a wide excision reduces the risk of local recurrence, it implies a more complex reconstruction. While skin grafting is technically straightforward, it may be inadequate when bone is exposed, due to poor adherence and limited coverage capacity, such as the distal phalanx of the big toe [22]. Therefore, a flap is often preferred to ensure adequate soft-tissue coverage and to protect the underlying anatomical structures (bone, joints, ligaments, and tendons). It was demonstrated that there was no statistically significant difference in local recurrence rates between wide excision with nail removal (4.7%) and amputation (2.9%) [23]. In this way, a flap reconstruction avoids amputation of the hallux, ensuring better post-surgical functionality.

Among the various types of flaps available, choosing a skinny one is advantageous [24]. Moreover, they sometimes require a second degreasing operation [24]. A thin fasciocutaneous pedicled flap is an advantage for reconstructing the first toe. Indeed, patients operated on with thick flaps frequently have difficulty putting on their shoes. One of the advantages of the pedicled flap based on the FDMtA is that it avoids complications related to vascular micro-anastomosis surgery. Lee et al. reported 41 cases of reconstruction following wide excision of the nail apparatus for melanoma using ultra-thin superficial circumflex iliac artery perforator (SCIP) flaps [25]. In this context, free flaps remain marginal due to the technical expertise required and the longer operative time involved.

Its simplicity, low morbidity, and favorable aesthetic outcomes make us prefer this flap over more complex free flap techniques or local options that may result in bulkier reconstructions. This anatomical study further supports its reliability and provides a detailed guide to its anatomy and surgical application. In our clinical practice, this flap is used as the first-line approach for reconstruction of the hallux, particularly in oncologic cases such as melanoma.

A correct dissection and isolation of the FDMtA perforator is necessary to allow this flap to rotate up to 180°, making this propeller flap the most adequate in the lower extremities [15]. As described for distal tibial defects, it seems that a 180° propeller flap provides a more esthetic result and facilitates direct closure of the donor site [26].

Among the techniques described for repairing toe defects, we can highlight the dorsalis pedis fasciocutaneous flap (DPA), which provides good distal reconstruction [22]. However, it presents risks of complications at the donor site, such as poor skin graft uptake, ulceration, and flap necrosis [22]. A similar technique, such as the lateral tarsal flap with a reverse dorsalis pedis artery pedicle, also presents risks of necrosis or severe venous insufficiency [27].

This anatomical study’s cartography confirms the flap’s abundant vascularization, thanks to numerous perforating arteries (FDMtAP) with about 3.67 perforators per foot, grouped over 8 cm^2^, making it viable and reliable. Analysis of their tiny diameter (oscillating between 0.10 mm distally and 0.25 mm proximally) suggests that dissecting them would be too hazardous. We advocate that it is safer to preserve a couple of perforator branches when harvesting the flap by maintaining the fatty area from which they emerge. This information is essential, as it saves precious time during pre-surgical identification of the FDMtA and its cutaneous perforators. Regarding preoperative flap mapping, we do not recommend the use of CTA, MRA, or ICG imaging. For a trained surgeon, acoustic Doppler or ultrasound is sufficient to identify the perforator.

Moreover, its proximity to the receiving site makes it ideal from an aesthetic point of view. It has the advantage of being smaller and more distal than the flaps currently used [9,28]. The donor site with an artificial dermis does not require skin grafting and avoids its associated morbidity. The donor site covered with artificial dermis does not necessarily require skin grafting, and we intentionally avoid skin grafts to minimize associated morbidity.

The main limitation of our study is the inconclusive vascular territory analysis. Indeed, the small size of the perforators did not allow us to correctly study the flap perfusion area after selectively catheterizing the perforator for red ink injection. Finally, our anatomical study presented a limited number of dissections. Nevertheless, the small standard deviation in the statistical analysis allowed significant conclusions, and no further dissections were performed.

## 5. Conclusions

This anatomical study of FDMtAPs highlighted the presence of a constant and rich dorsal cutaneous vascular network in the foot. The results of the surgical technique are satisfactory, both functionally and aesthetically. The first dorsal metatarsal artery perforator flap seems to be a reliable solution for the hallux soft-tissue reconstruction, notably after the excision of acral melanoma.

## Figures and Tables

**Table 1:** Results of the FDMtAP measurements. FDMtAPs: first dorsal metatarsal artery perforators; SD: standard deviation; L = left; R = right; f = foot.

Analysis of the First Dorsal Metatarsal Artery Perforators (FDMtAPs)						
Specimen No.	Foot (n)	Side	Mean Number of FDMtAPs (n)	Mean Length of FDMtAPs (cm)	Mean Proximal Diameter of FDMtAPs (mm)	Mean Distal Diameter of FDMtAPs (mm)
1	f1	L	5	2.94	0.17	0.11
2	f2	R	6	2.42	0.13	0.11
2	f3	L	3	2.07	0.16	0.1
3	f4	L	3	1.94	0.16	0.1
4	f5	L	3	1.96	0.19	0.12
4	f6	R	4	2.07	0.22	0.12
5	f7	R	4	2.42	0.16	0.1
6	f8	R	2	2.75	0.25	0.12
7	f9	L	3	2.53	0.16	0.11
Mean	—	—	3.67	2.35	0.178	0.110
SD	—	—	1.23	0.36	0.037	0.008

## Data Availability

Data can be provided by the corresponding authors on demand.

## Abbreviations

The following abbreviations are used in this manuscript:

FDMtA	First dorsal metatarsal artery
FDMtAP	First dorsal metatarsal artery perforator
DPA	Dorsal pedis artery
HFM	Head of the first metatarsal
BFM	Base of the first metatarsal
ATA	Anterior tibial artery
IHC PRAME	Immunohistochemistry preferentially expressed antigen in melanoma
Tis	Tumor in situ
AJCC	American Joint Committee on Cancer
